# An Overview on Principles for Energy Efficient Robot Locomotion

**DOI:** 10.3389/frobt.2018.00129

**Published:** 2018-12-11

**Authors:** Navvab Kashiri, Andy Abate, Sabrina J. Abram, Alin Albu-Schaffer, Patrick J. Clary, Monica Daley, Salman Faraji, Raphael Furnemont, Manolo Garabini, Hartmut Geyer, Alena M. Grabowski, Jonathan Hurst, Jorn Malzahn, Glenn Mathijssen, David Remy, Wesley Roozing, Mohammad Shahbazi, Surabhi N. Simha, Jae-Bok Song, Nils Smit-Anseeuw, Stefano Stramigioli, Bram Vanderborght, Yevgeniy Yesilevskiy, Nikos Tsagarakis

**Affiliations:** ^1^Humanoids and Human Centred Mechatronics Lab, Department of Advanced Robotics, Istituto Italiano di Tecnologia, Genova, Italy; ^2^Dynamic Robotics Laboratory, School of MIME, Oregon State University, Corvallis, OR, United States; ^3^Department of Biomedical Physiology and Kinesiology, Simon Fraser University, Burnaby, BC, Canada; ^4^Robotics and Mechatronics Center, German Aerospace Center, Oberpfaffenhofen, Germany; ^5^Structure and Motion Laboratory, Royal Veterinary College, Hertfordshire, United Kingdom; ^6^Biorobotics Laboratory, École Polytechnique Fédérale de Lausanne, Lausanne, Switzerland; ^7^Robotics and Multibody Mechanics Research Group, Department of Mechanical Engineering, Vrije Universiteit Brussel and Flanders Make, Brussels, Belgium; ^8^Centro di Ricerca “Enrico Piaggio”, University of Pisa, Pisa, Italy; ^9^Robotics Institute, Carnegie Mellon University, Pittsburgh, PA, United States; ^10^Applied Biomechanics Lab, Department of Integrative Physiology, University of Colorado, Boulder, CO, United States; ^11^Robotics and Motion Laboratory, Department of Mechanical Engineering, University of Michigan, Ann Arbor, MI, United States; ^12^Department of Mechanical Engineering, Korea University, Seoul, South Korea; ^13^Control Engineering group, University of Twente, Enschede, Netherlands

**Keywords:** variable impedance actuators, energy efficiency, energetics, cost of transport, locomotion principles, bio-inspired motions

## Abstract

Despite enhancements in the development of robotic systems, the energy economy of today's robots lags far behind that of biological systems. This is in particular critical for untethered legged robot locomotion. To elucidate the current stage of energy efficiency in legged robotic systems, this paper provides an overview on recent advancements in development of such platforms. The covered different perspectives include actuation, leg structure, control and locomotion principles. We review various robotic actuators exploiting compliance in series and in parallel with the drive-train to permit energy recycling during locomotion. We discuss the importance of limb segmentation under efficiency aspects and with respect to design, dynamics analysis and control of legged robots. This paper also reviews a number of control approaches allowing for energy efficient locomotion of robots by exploiting the natural dynamics of the system, and by utilizing optimal control approaches targeting locomotion expenditure. To this end, a set of locomotion principles elaborating on models for energetics, dynamics, and of the systems is studied.

## 1. Introduction

The recent development in design and control of active or intrinsically controlled Variable Impedance Actuators (VIA) has demonstrated remarkable advancements in safety, robustness and peak power performance. However, despite the above, considerable performance improvements and progress made in the past 20 years in mechatronics and control, the motion/locomotion efficiency of even the most energy efficient robots still remains many times smaller than that of humans or animals. Due to these deficiencies there are several untethered applications (humanoids, manipulators, assistive and power augmentation exoskeletons, prostheses) where the limited power autonomy prevents their full practical exploitation. The advancement on robotic economy will therefore substantially impact the viable exploration of robotics in all applications requiring untethered operation. To enable this progress, new design principles and technologies are needed.

This paper^1^ reviews the recent advancements in new robot design principles and control that target to reduce their energy consumption and lead to robots that are more efficient. The main objective is to present an overview of recent activities on the development of these new robot machines. Emerging topics to improve energetic performance of these robotic systems include novel joint-centralized variable impedance actuation, energy neutral intrinsic load cancellation and lock/release mechanisms, variable recruitment actuation principles, bio-inspired distributed compliance actuation, embedded energy buffering and high efficiency power transmission concepts. The exploration of control principles for effective energy recycling and load cancellation during motion is also fundamental for improving the energy efficiency of these machines and will also be discussed with an emphasis on techniques for the exploitation of intrinsic resonance modes and energy efficient impedance regulators for under-actuated variable impedance or multi-articulated robots. Contributions on the biological side, particularly on the energy economy of humans and animals as well as on the biomechanics of locomotion efficiency, are complimentary and will provide the ground reference for today robot efficiency as well as set the energy efficiency goal of future robotic machines. In summary, we aim at demonstrating recent developments in mechatronics and control with a focus on the energy economy of robotic systems, to advance the understanding of actuation and control principles contributing to energetic economy in biological systems, humans and animals.

To clarify the energy efficiency of current robotic systems, we compare the Specific Resistance^2^ (SR) of biological systems with that of robots. For instance, a horse trots with a SR of 0.2, and humans walk with a similar SR of 0.2. Based on the data reported by Tucker (1975) for Cost of Transport (CoT) of humans and animals, passive walkers (see the work by McGeer, 1990) are considerably more efficient than humans as shown by Collins et al. (2001), although they possess very limited flexibility in terms of functioning versatility and demand carefully dynamics tuning of operating conditions e.g., initial states. Humanoid robots that are capable of replicating human-like motions and executing human tasks, however, render fairly larger CoT/SR as compared to humans: Asimo presented by Sakagami et al. (2002) exhibits an SR of 2 (1.8 KW for 1.5 m/s, with a mass of 54 kg) and Durus introduced by Reher et al. (2016) targets an SR of 1. These are amongst the most efficient humanoids robot while the CoTs of them are ten and five times larger than human CoT, respectively. Similarly, the hydraulically actuated Big Dog operates with an SR of 15 that limits the autonomous operation time to about 30 minutes given the fuel capacity limitation (15 L of fuel for 20 Km, with a mass 110 kg). On the other hand, mammalian skeletal muscle exhibits a power density of 0.041 W/g and has about a 25% efficiency for concentric muscle action, while a motorized actuator can render higher values. An individual motor can possess a power density of 0.5 W/g and about 80% efficiency, although theses values may drop to 0.17 W/g and 40% when combined with a typical gearbox, respectively. Yet, the MIT cheetah actuators that exploit electrical energy regeneration/recycling demonstrate an SR of 0.5.

To understand efficient motion, we analyse human walking/running. Human locomotion comprises mostly unforced motion, where back-drivability significantly enhances the efficiency, and presents considerable energy storage due to recycling. Power consumption of the Walk-Man robot developed for performing disaster response tasks as the primary target, introduced by Tsagarakis and et al. (2017) requires about 387 W for electronics (45 W for perception system, 62 W for two processing units, and 280 for 36 motor driver electronics), and the total power consumption for standing is about 420 W. Slow walking (20 cm per second) requires a total power of 510–755 W in the most demanding condition. This shows that the maximum consumption includes an actuation power of 368 W; thereby representing an SR of 1.35. This describes an SR seven times higher than that of human walking only for actuation, while the total consumption expresses an SR of 2.8 which is 14 times larger than that of human walking. However, the lack of efficiency in comparison with humans is expected as the energy storage capacity of the system is limited to passive compliant elements with small deflection, that leads to large energy consumption for moving/accelerating joints. The other significant cause is the high gearing that renders large reflected inertia and results in a strict need for forced motions.

**Figure 1:**
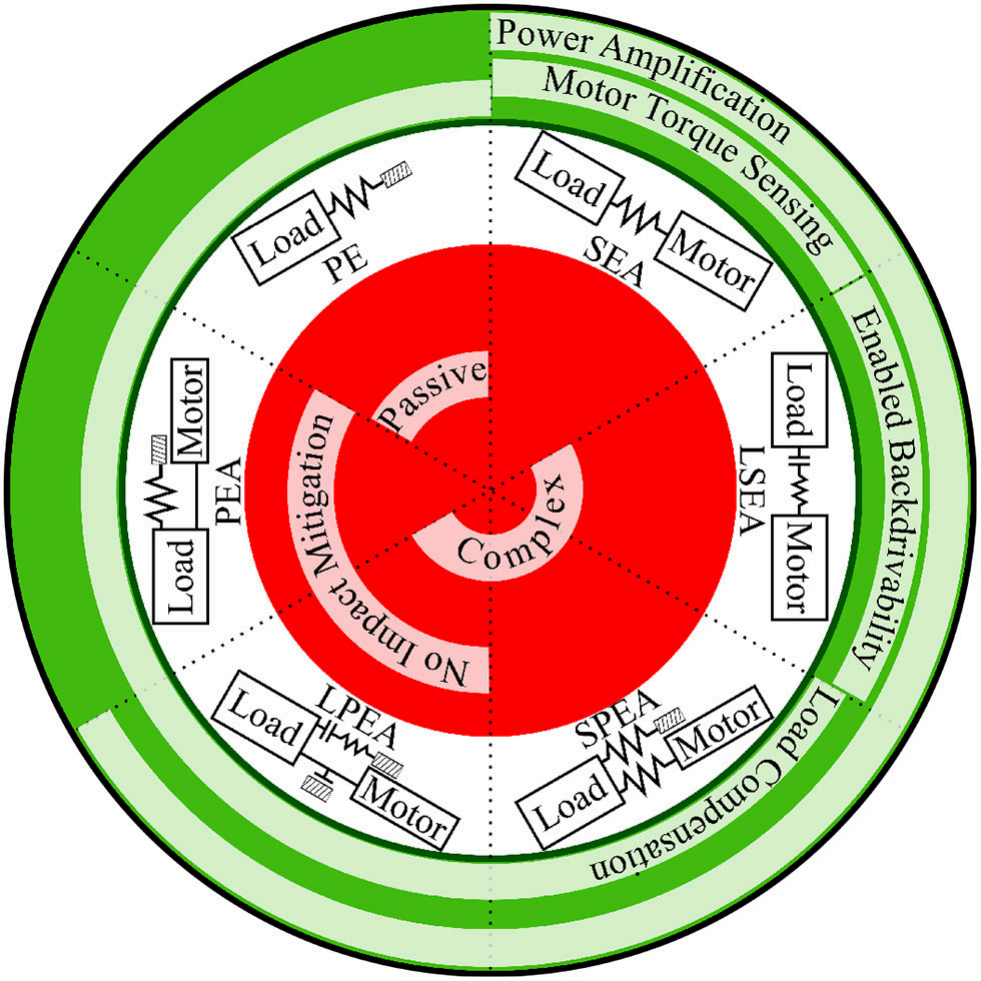
A set of typical implementations of compliance in robotic joints, with main pros and cons shown in green and red respectively.

By incorporating the passive dynamics, as well as kinematic and actuator optimizations, the energetics performance of the robot can be significantly improved. Preliminary experiments on the bipedal robot Cassie show that the 30 kg robot can walk at 1.0 m/s using a total of 200 Watts of power while performing different locomotion behaviors such as squatting, thereby rendering an SR of 0.7. This efficiency is owed to not only added compliance, but also to a leg design that (i) selects actuator/transmission through a joint-level actuator work minimization for performing walking tasks, see Rezazadeh and Hurst (2014); (ii) designs minimal toe inertia to reduce ground impacts, see Abate et al. (2015); (iii) utilizes a leg kinematics configuration which balances net task power among involved actuators (see Abate et al., 2016). Advancement in energy efficiency of robotic systems requires attention in various aspects of the robot operation problem, ranging from actuation and limb design to motion control. Table 1,^3^ presents a comparison between the energetics of biological systems and current robotic systems. This paper reviews recent advancements in design and control of robots, to elucidate the energetic state of current robotic and mechatronic systems in comparison with biological systems, and to derive insights and features for the development of more energy efficient robots. The rest of the paper is structured as follows: section 2 reports the variety of compliant actuators propounded for enhancing energy efficiency of robots, (see Figure 1). Section 3 discusses the importance of limb segmentation in design, dynamics and control, and how the robot design should rely on this information. Section 4 discusses a set of state-of-the-art energy efficient control methods on the basis of bio-inspired and optimal control principles. Section 5 describes a set of cutting-edge concepts initiating novel directions for robot locomotion. Finally, the paper summary is described in section 6.

**Table 1:** Energetics comparison of biological and robotic systems.

**Criteria**	**Biological systems**	**Robotic systems (2017)**
Actuator Power Density	500 W/kg (Muscle)	200–300 W/kg (BDC+Harmonic Drive)
Actuator Energy Efficiency	20% (Muscle)	40–50%
Computation Power	20 W (Human Brain)	60 W (a regular notebook)
Power Consumption at Rest	60–80 W (Basic Metabolism)	150–400 W
Energy Storage	17 MJ/kg (Carbs)	0.87 MJ/kg (Li-Ion)
	Complicated digestion mechanism	Efficient and lightweight power converters

## 2. Compliant Actuation

Despite the potential of most electric motors for electrical energy regeneration, it is not often used in robotic systems, except for highly dynamic robots using direct drives e.g., cheetah robot presented by Seok et al. (2015). The lack of use is likely due to substantial losses in high gear reduction systems typically used in robotics requiring high torques, as discussed by Verstraten et al. (2015), thereby rendering significant copper losses (heat produced by electrical currents in motor windings) and leading to trivial energy regeneration. Furthermore, the actuation systems typically suffer from having the link and motor in a series chain. As a result, all torques pass from the motor, and even in stationary conditions, i.e., zero mechanical power, there is a non-negligible electrical energy consumption. Mechanical storage mechanisms/methods are therefore exploited in robotic machines, which often rely on integration of passive elastic elements into actuation units. Nevertheless, a suitable choice of a mechanical storage system cannot be based only on the mechanical energy domain. As discussed in Verstraten et al. (2016), the dynamics of reduction system and motor dynamics, as well as the operating position and range of motion, influence the optimal compliance and whether series and/or parallel compliance is more beneficial. As shown in Beckerle et al. (2017), while the use of compliance in series with the motor can often be more energy efficient, compliance in parallel may become more beneficial when the operating point changes (therefore more static force).

As animal structure and motion have simultaneously evolved to be specifically designed to perform desired tasks efficiently and effectively, it is essential to account for both morphology and motion in robot design and control. Yesilevskiy et al. (2015, 2018a) discussed the effect of morphological variations in legged robots, and showed for a one-dimensional monoped hopper that is driven by a geared DC motor, with the correct choice of the transmission parameter, a hopper with Series Elastic Actuators (SEA) is more energetically efficient than one with Parallel Elastic Actuator (PEA). It is mostly due to the fact that, for a hopper with PEA, the motor inertia contributes to energetic losses due to the ground contact collisions. The energy saving capacity of this kind of compliance arrangement had been shown not only when used in legs, but also when used in torso structure as shown by Folkertsma et al. (2012). In all these cases, the natural mechanical dynamics connect the motion of a robot or animal to its morphology, thereby inherently coupling control and design of such systems.

### 2.1. Compliance in Series

The incorporation of built-in compliance into robot joints was primarily introduced to enhance the shock tolerance capacity via generation of inelastic collision force spikes, system responsivity due to higher force control bandwidth, and energy efficiency by cycling the energy flow; as well as amplifying output power. To address the limited storage capacity of the passive compliant component, various designs enabling larger deflection were proposed, e.g., by Tsagarakis et al. (2009). As presented by Verstraten et al. (2016), the inclusion of compliance in parallel or in series allows a decrease in both peak power and energy consumption provided that the stiffness of the elastic element is tuned properly, and substantiates the development of variable stiffness actuators, see Vanderborght et al. (2013).

Nevertheless, compliant systems exhibit unwanted vibrations that require inclusion of active, semi-active and passive damping. While active impedance control has been widely studied, e.g., by Ferretti et al. (2004); Kashiri et al. (2014b), the active dissipative action is often limited by the control-loop bandwidth, and suffers from feedback noise and phase-lag problems. The semi-active and passive damping therefore proved to be more effective as presented by Laffranchi et al. (2014) and Kashiri et al. (2017a). However, the inclusion of damping amplifies the complexity and total mass of the system. The former escalates the development and maintenance costs, and the latter reduces the energy efficiency. Moreover, semi-active solutions require additional controllers that intensify the complexity of the system, e.g., see works by Kashiri et al. (2014a), Kashiri et al. (2015), and Kashiri et al. (2016). As a result, despite the proven energy consumption reduction achieved by such systems, the utilization of variable impedance solutions, when considering the additional hardware/software complexity, requires careful attention to the application requirements.

### 2.2. Compliance in Parallel

#### 2.2.1. Asymmetric Configuration

To develop a robotic actuator that combines high power and physical robustness with energetic efficiency, Tsagarakis et al. (2013) proposed an asymmetric compliant antagonistic joint concept, and developed a 1-DOF knee-actuated prototype leg. The novel design featured variable quasi-static load compensation, moderate gearing, large energy storage capacity, and controllable energy storage/release. It includes two branches working in parallel to each other: a high power branch, and an energy storage branch. The first branch embodies a series elastic actuation system, with an elastic element serving as a bi-directional coupling between the drive and the output link, protection of the drive unit, and torque sensing as demonstrated by Kashiri et al. (2017b). The second branch includes a low power motor coupled with a high reduction efficient linear transmission in series with a passive elastic transmission, and an elastic element with large energy storage capacity. To achieve this, it uses an elastic band similar to bungee cords, instead of metal springs. The coupling between the low power drive and the elastic element is done using a non-backdrivable transmission component, and a two-way overrunning clutch module, to remove the effort for maintaining pretension of the elastic element. On the basis of this concept, Tsagarakis et al. (2014) demonstrated the efficiency benefits of the large energy storage capacity in cyclic motion operations and in static load compensation in a unit with Series Parallel Elastic Actuator (SPEA). Roozing et al. (2016b) further expanded the work and proposed an integrated control strategy that actively utilized both branches, and experimentally demonstrated a 65% reduction in electrical power consumption when compared to conventional SEA.

Roozing et al. (2018) presented the development of a semi-anthropomorphic 3-DOF leg design, where series elastic actuation units are complemented with parallel high efficiency energy storage branches. Based on the earlier work in Roozing et al. (2016a), three exchangeable actuation configurations were described, where the energy efficiency achieved by parallel elasticity branches in mono- and bi-articulated configurations is demonstrated, as compared to a series-elastic-only configuration as a baseline. In biomechanical systems, biarticulated muscles span multiple joints and thereby allow the transfer of mechanical power between joints. Similarly, in the bi-articulated actuation configuration, the energy storage branch allows the transfer of mechanical power between the robotic knee joint and ankle joint. The multi-DOF energy storage branch system can thus provide a desired torque profile over the range of motion in an efficient manner to obtain minimal energy consumption and/or maximum gravity compensation. Additionally, it can increase peak torque output and assist in explosive motions such as push-off during running. The leg presented in Roozing et al. (2018) performed a set of squat motion in three different configurations: without parallel elastic storage, and with parallel elasticity in mono-articulated and in bi-articulated configurations, with overall masses of 7.57, 9.14, and 9.21 kg, respectively, and exhibiting 33.1, 15.4, and 13.2 W electrical motor power when lifting 20 kg by 25.6 cm. On the other hand, humans^4^ consume approximately 41, 53, and 54 W to perform a similar squat motion, for the overall mass of afore-mentioned configurations. If we account for efficiency of actuation in both cases (20% in muscles and 40% in robotic drives), the mechanical output power of the robotic leg is 13.2, 6.2, and 5.3 W, while that of humans is 8.2, 10.6, and 10.8 W; confirming the importance and effectiveness of parallel elasticity units.

#### 2.2.2. Symmetric Configuration

Due to a strong demand for energy-efficient, yet low-cost, robotic arms, it is necessary to minimize the torque required to operate a robot while maintaining high performance. Considering robots developed for interaction purposes often operate at low speeds, the dominant torque is to carry the robot weight, especially when the payload to robot mass ratio is low. Gravity compensators can therefore save a significant amount of mechanical energy. It is therefore beneficial to counterbalance the gravitational torques resulting from robot mass, and accordingly employ the motor efforts for gravitational torque of varying payload and the inertial torques. Despite several advancements toward such efficient robotic manipulators using spring-based counterbalancing, the majority of such systems are often bulky and heavy, with a small range of rotation, and their utilization is limited to a one or two DOFs, see designs proposed by Koser (2009), Nakayama et al. (2009), and Lacasse et al. (2013). Passive gravity compensation using a counterbalance spring-based mechanism can rely upon various concepts, including (1) Wire-based systems, e.g., the service robotic arm proposed by Kim and Song (2014); (2) Gear-based devices; (3) Linkage-based mechanisms, such as the slider-crank based system developed by Kim et al. (2016). Given a multi-DOF manipulator, it is necessary to deal with continuously varying gravitational torques that depend on the robot configuration, thereby increasing the need for multi-DOF counterbalancing.

A possible solution to this problem is the development of a mechanism with a reference plane corresponding to each link, so that the spring counterbalancing the gravitational torques connects the link to this reference plane, and accordingly compensates for the gravitational torque of each link independently. Such a reference plane therefore is required to automatically align with the gravity direction, and enables proper linking with neighboring reference planes in a way that the compensated torque is transmitted to the ground, and the motion of one link does not affect others. On the basis of this concept, Korea University developed a series of spatial robots with multi-DOF counterbalancing features: including SCORA-H, SCORA-V, and KU-WAD. The latter employs a counterbalance mechanism based on two sections: (1) Spring and wire: to generate the compensation torque exhibiting zero counterbalance error; (2) Timing belt and pulley: to provide a suitable reference plane interconnection, and render a wide operation range without dead points. Experiments on SCORA-V (with six DOFs, 49 kg mass and 8 kg payload capacity) show significant mechanical and electrical energy savings thanks to the mechanical gravity compensation feature. Without using the gravity compensation system, the robot requires about 160 W for maintaining a high gravity posture and consumes 170 Wh for 1 h of operation (a repetitive task), while these values reduce by 34 and 30% by exploiting the counterbalancing mechanisms, respectively. Such a reduction in electrical energy consumption enables the use of smaller motors and gearing transmission systems in the design process, and saves a considerable amount of energy in the long-term use of a robot.

### 2.3. Compliance in Series/Parallel With Locking Mechanism

Another solution addressing actuation inefficiencies was presented by Plooij et al. (2016); Geeroms et al. (2017), that exploits Lockable Parallel Elastic Actuator (LPEA), as well as series elasticity. Experiments on a knee prosthesis powered by this actuator showed an energy consumption of 65 J/stride and peak power of 100 W, which are considerably lower than those generated by the same system powered by a direct drive (400 W and 156 J/stride) and SEA (200 W and 98 J/stride). Mathijssen et al. (2016) proposed another potential solution: the use of redundancy in actuation when a set of lockable SEAs (LSEAs) are set in parallel, to show a clear difference in mechanical energy consumption due to parallel springs that can be loaded with respect to any desired position. However, Verstraten et al. (2018) discussed the advantages of exploiting actuation redundancy in a more generic case, i.e., a multi-motor drive as compared to a single drive, to present smaller size and mass, and more importantly, a considerably higher efficiency for slower speeds, and larger static operation range; although it shows lower maximum efficiency, and slightly lower bandwidth. Mathijssen et al. (2017) discussed a similar concept for development of a discrete muscle-inspired actuator, presenting how this approach allows overpowering binary drive units (solenoids).

Chen et al. (2013) presented the employment of a locking mechanism in parallel with compliance in series, in order to store and release energy at the right time. Malzahn et al. (2018) discussed the employment of locking mechanisms (clutch) in series with the drive train possessing compliance in series, i.e., Lockable SEA (LSEA). The work is inspired by human muscle activity in different phases of running. As presented by Novacheck (1998), while the stance phase requires highly active motion of the joints to overcome gravitational torque, motion of the joints during the swing phase is passively driven by gravitational and inertial link torques. It is therefore more efficient to relax the muscles during the swing phase of running and exploit the passive dynamics of the system. However, the high transmission ratios used in conventional electric robot actuation do not permit such an operation. The transmission ratio trades the high speed of electric drives for increased output torques, which enable high torque density actuator designs. But, the transmission ratio steps up the motor as well as gearing friction torques, which render the actuation barely backdrivable by gravitational and inertial torques. Unlike in the human example, the motor therefore has to actively drive and overcome the intrinsic friction throughout any motion phase. As an alternative to direct drives, a clutch mechanism in series between the transmission and the link can be utilized so that the conventional mature high torque density drive unit can be partially/fully disengaged when the passive dynamics can partially/fully generate the required motion. The energy saving potential of the series clutch approach depends on the motion dynamics and the ratio between the link gravitational and motor friction torques. The theoretical savings range between 20 and 60 % of the mechanical energy required to perform the same motion compared to the identical drive without series clutch actuation.

### 2.4. Purely Passive Compliance

While robotic platforms often utilize active joints, with and/or without Passive Elasticity (PE) integrated as discussed above, fully passive compliant joints are not often used except for end-effectors such as feet. However, they are commonly used in leg prostheses, where metabolic and biomechanical effects of people with lower extremity amputations is of utmost importance. Herr and Grabowski (2012) presented a powered prosthesis (BiOM) that enables people with a transtibial amputation to achieve normative metabolic costs, preferred walking speeds, and step-to-step transition work while walking over level ground across a wide range of speeds. D'Andrea et al. (2014) showed that use of the BiOM enhances the regulation of whole-body angular momentum, and therefore reduces fall risk. Grabowski and D'Andrea (2013) found that use of the BiOM reduces unaffected leg knee loading and thus osteoarthritis (OA) risk. Jeffers et al. (2015) compared changes in metabolic power and mechanical power during step-to-step transitions while non-amputee subjects walked on a range of slopes at different velocities. They found that at faster velocities, metabolic power increased, the leading leg absorbed more power, and the trailing leg generated more power compared to slower velocities. Moreover, with increasing slopes, mechanical work of the leading leg became more negative while mechanical work of the trailing leg became more positive. Jeffers and Grabowski (2017) also found that use of the powered prosthesis (BiOM) compared to a passive-elastic energy storage and return (ESAR) prosthesis, improves biomechanics and metabolic cost on uphill slopes.

To discuss the effects of compliance and geometry of a class of passive-elastic running-specific prostheses in series with the leg, a set of this class of prostheses developed by manufacturers based on subjective stiffness categories was studied. Beck et al. (2016) found that the prosthetic stiffness values of these manufacturer recommended stiffness categories varied between prosthetic models. They also found that the force-displacement profiles of such prostheses are curvilinear, indicating that prosthetic stiffness varies with the magnitude of applied force. Beck et al. (2017) investigated the effects of running-specific prosthetic stiffness, height and speed on the biomechanics of a set of athletes with bilateral transtibial amputations. They found that with use of stiffer prostheses, athletes could apply greater peak and stance average vertical ground reaction forces, increase overall leg stiffness (inversely associated with running speed), decrease ground contact time and increase step frequency; although these effects were reduced at faster running speeds. The effects of ±2 cm changes in prosthetic height on biomechanics (inversely associated with step frequency) were unchanged. It was also shown that J-shaped running-specific prostheses often outperform C-shaped prostheses in terms of both metabolic CoT and maximum speed in athletes with transtibial amputations.

## 3. Segmented Limbs

Periodic motions such as crawling, walking, and running, are typical tasks in which energy storage and release frequently occur; where muscle and tendon elasticity plays the most important role in biology. Efficient generation of such motions requires realization and characterization of periodic oscillations. Eigenmodes of linear dynamics have been widely used for rendering such motions based on the classic generalized eigenvalue problem (see works by Blickhan, 1989; Geyer et al., 2006; Kashiri et al., 2017c). To approach the performance and efficiency of the biological archetype, it is crucial to employ physical elasticity in drive units. To exploit the full dynamics of segmented legs, Lakatos et al. (2017) described eigenmodes of non-linear dynamics. Such a complete modal model can enhance the system performance as the robot design targets match the segmented leg dynamics with a desired dynamics set based on template models, e.g., Spring-Loaded Inverted Pendulum (SLIP), and desired motion considerations. While the robot structure design relies primarily upon geometry specifications, statics requirements, and actuation principles; design of kinematics, elasticity and inertial parameters respecting the above-said matching dynamics initiates a mechanical system with an embodied modal task, as discussed by Duindam and Stramigioli (2005). Such a design can significantly facilitate the robot control; similar to humans who excite the resonance by means of timed and directed motions.

Geyer et al. (2006) discussed the significance of segmented leg dynamics, which are often ignored, thereby creating untapped potential for improved mechanical energetics and control. Exploring point mass models whose legs are reduced to force laws shows that increasing the number of leg segments helps to reduce the mechanical advantage of leg force with less burden on joint actuators, i.e., lower joint torques; however, it also increases the design complexity. In addition, multiple segments create internal degrees of freedom, which introduce joint buckling in elastic stance leg behavior. Seyfarth et al. (2001) showed different strategies to mitigate this destabilizing effect from segment length changes (non-equal lengths) to non-linear joint elasticities to bi-articular actuation. Such remedies enlarge the design complexity. In addition, studies on human locomotion revealed the swing leg dynamics are double-pendulum like and appear nearly passive, as reported by Mochon and McMahon (1980). However, the motion of a double-pendulum is chaotic if not properly restrained. Potential field calculations of the foot point (using energy neutral coupling springs) show that the double pendulum can be suitably restrained by passive bi-articular coupling, resulting in natural and comparably robust swing leg behavior. Overall, these two examples show leg segmentation introduces significant challenges to locomotion dynamics, which if they are ignored, lead to increased control effort and actuator energy expenditure.

The knowledge gained from studying the effects of leg segmentation can benefit the control of robotic limbs. Desai and Geyer (2012, 2013) showed the influence of incorporating active bi-articular coupling into nonlinear swing leg control to generate natural swing leg motions without pre-recorded reference trajectories. Moreover, the resulting control approach can position the leg into a wide range of target postures with robustness to large swing disturbances. On the basis of this concept, Thatte and Geyer (2016) formulated a control policy for powered knee-ankle prostheses. Simulation results suggest this policy generates human-like leg behavior in steady walking, and responds to disturbances to the swing leg with experimentally observed elevation and lowering strategies. Furthermore, a comparison with the performance of an impedance controller shows the proposed policy enables a computer model of an amputee to walk over rough terrain and recover from larger disturbances.

## 4. Energy Efficient Control

Efficient motion of robotic systems can be based on various principles. A majority of studies in this area employ optimal control techniques and bio-inspired approaches. Another paradigm for the control of such systems is energy-aware robotics that targets energy flows, especially in interactions. This provides a basis to constructively tackle issues of stability during interaction and methods to analyse energy storage and consumption in robotics systems. Stramigioli (2015) and Folkertsma and Stramigioli (2017) elaborated the basis of this concept to present a universal framework that models drive and interacting robotic systems, as the basis for energy-limited control, so that the actuation controllers can expend an energy budget to execute a given task, without injecting more energy. Below, we discuss a set of state-of-the-art optimal/bio-inspired approaches.

### 4.1. Natural Motion

Development of systems capable of executing efficient cyclic motions requires the exploitation of the robot's natural dynamics (McGeer, 1990; Collins et al., 2005; Ferris et al., 2007). Mechanisms for efficient natural dynamics have often been inspired by studies of the agile and efficient dynamics of human and animal locomotion (McMahon, 1985; Ferris et al., 1998, 2007; Full and Koditschek, 1999; Daley and Biewener, 2006; Geyer et al., 2006). A bio-inspired Central Pattern Generator (CPG) approach is one commonly-used method for rendering cyclic motions, as explored by Ijspeert (2008). However, the method relies upon an isolated unit to generate a periodic motion pattern. The control structure is thus open-loop as the controlled system feedback is not considered in the control. Furthermore, the oscillatory dynamics of the elastic body (robotic or biological) system are not exploited. To identify/realize the resonance excitation mechanism of humans, Lakatos et al. (2014) carried out a set of psycho-physical experiments including a human in the control loop; when the human is asked to excite a simulated elastic limb to a limit cycle in minimum time or with minimum effort, and the force feedback device displays to the human the forces from simulation. To estimate human adaptability, the limb parameters are arbitrarily varied in simulation. The results revealed a bang-bang control law switching the position reference around an equilibrium point when the force feedback changes direction. Lakatos and Albu-Schäffer (2014b) exploited this switching control approach for generating limit cycle motions. In order to excite a multi-dimensional, non-linear elastic multi-body system, Lakatos and Albu-Schäffer (2014a) proposed to apply the bang-bang controller in the torque transformed direction, and compute the corresponding reference joint positions from the modal coordinate. Lakatos et al. (2014) showed the functionality of this approach in generating cyclic motions on a DLR variable stiffness arm, which can elaborate the modal coordinate transformation from a neuroscience perspective as “dynamics synergies”, see the work of Stratmann et al. (2016).

### 4.2. Minimal Energetics

Legged locomotion in nature can be observed to happen in a variety of different gaits that can be characterized by their different footfall patterns and contact forces, as discussed, for example, by Hildebrand (1989). Biomechanical experiments have established a clear relationship between running speed, choice of gait, and energy consumption in humans by Minetti and Alexander (1997) and horses by Hoyt and Taylor (1981). These data suggest that animals may change gait as a function of locomotion speed based on metabolic CoT. To capture the effect of gait in robotic systems, Xi and Remy (2014); Xi et al. (2016) employed optimal control for motion generation of conceptual models of bipeds and quadrupeds. The approach generated motions that minimized positive mechanical work (normalized by distance traveled) while being subject to realistic robot dynamics and locomotion constraints such as foot non-penetration or actuator limits. By varying forward speed and contact sequence, the results show that changing gait as a function of locomotion speed can substantially increase mechanical economy. The optimal behavior in bipedal locomotion is to walk at slow speeds and run at high speeds and in quadrupedal robotic locomotion to walk at slow speeds, trot at intermediate speeds and gallop at high speeds. It is notable that there was only a small mechanical energetic difference between trotting and toelting, which may explain why the toelt is part of locomotion repertoire of some horses. In contrast to biological quadrupeds, galloping did not significantly outperform trotting in simulations. This might be attributed to the lack of an articulated spine in the original quadrupedal model Yesilevskiy et al. (2018b).

Smit-Anseeuw et al. (2017) extended this approach to discuss optimal motions for the bipedal robot RAMone, and investigated the results of comparing two different footfall sequences (a walking sequence with a double support phase and a running sequence with aerial phase) and two different orientations of the knee joints (pointing forwards and backwards). It showed the optimal gait switches from ballistic walking with an instantaneous double-support to spring-mass running with an extended aerial phase at a speed of around 1 m/s. That is, at slow speeds nearly no elastic energy is stored in the actuator springs, while at high speeds almost all of the mechanical energy fluctuations within the robot are conducted through the springs. Switching from ballistic walking to spring-mass running reduced metabolic energy consumption by up to 88%. This is comparable with studies on the metabolic cost of human walking.

Donelan et al. (2001) showed, when humans walk, they prefer a particular step width, and execute this preference with remarkably small variability. In arriving at this preference, the nervous system may seek to minimize an objective function composed of a weighted sum of objectives. One such objective may be metabolic cost. Toward understanding how the nervous systems of able-bodied people weight this objective in walking, Selinger et al. (2015) measured people preferred gait in different cost landscapes, defined as the relationship between metabolic cost and a given gait parameter, and demonstrated that people can continuously optimize step frequency to minimize metabolic energy. Abram et al. (2017) evaluated results on several able-bodied subjects and found that preferred step width in a new landscape was determined by continuous energy optimization. Using step frequency as that gait parameter, Selinger et al. (2016) found the key features that describe this energetic cost optimization process, which can also be partially reproduced using a simple reinforcement learning algorithm, as shown by Simha et al. (2017).

Human motor control relies on central loop control, synergies, learning and peripheral loop (reflexes) as core principles, while robot motion control for transport (locomotion) is typically based on a decentralized position/torque/impedance controller and motion generation via simple models such as cart-table and SLIP. Such classical methods cannot exploit the full-body robot dynamics to obtain efficient motions. A possible solution to address this problem can be to determine the robot trajectories based on whole-body dynamics using a trajectory optimization approach targeting minimum CoT. Gasparri et al. (2018) formulated an optimal control problem in a way that robot dynamic parameters such as joint impedance may also be optimized, in addition to typical state and control variables. Once the locomotion constraints defining periodic change of contact phases (single and double supports) are set, in addition to the robot dynamics and conventional constraints e.g., joint limits, the optimal control problem is solved using a direct method. It is, however, a computationally highly demanding problem that cannot be solved in real-time. To address this issue, a library of optimized trajectories is generated off-line, and then it is searched in real-time for the trajectory associated with the current robot states/conditions. Nevertheless, it is demanding to manage the trajectory library size for robotic hardware with a large number of DOFs. A feasible remedy to this problem is to decipher the trajectory library.

The method is applied to a six-Degree-Of-Freedom (-DOF) planar biped powered by compliant actuators. Based on Principal Component Analysis (PCA), 99% of the variance of 500 optimized trajectories can be explained by three principal components that can also be expressed/fitted by second order polynomials. The results of this implementation on various walking speeds render a CoT around 0.5, which is on average about five times more efficient than the CoT resulting from a Zero-Moment-Point- (ZMP-) based approach rendering the trajectory based on the cart-table model. Results show that the swing leg joint torques of the two techniques are comparable. However, the stance leg joint torques using the optimized locomotion are negligible while the ZMP-based locomotion render substantial joint torques due to the non-straight leg configuration. To realize the effect of compliance on CoT in walking/running, the optimization based approach is employed for two cases, when the robot is rigid and soft. The results show that the slow walking CoT in the two cases are similar, although the soft system exhibited considerably lower CoT for fast walking. In addition, the rigid system renders a CoT approximately twice as great as the soft system when running. Moreover, using the soft system reduces the walk-to-run transition speed, and increases the maximum feasible running speed.

## 5. Locomotion Principles

When comparing different animals in nature, as well as the above robotic optimization studies, it is remarkable that despite substantial differences in structure, legged systems of all kinds rely only on a small set of different gaits. One potential explanation could be that these gaits are a manifestation of the underlying mechanical natural dynamics of the legged system. Gan et al. (2016) explored this idea by reducing the models to be completely lossless. Even with such conservative models, all common bipedal and quadrupedal gaits can be represented as passive periodic orbits, suggesting that gaits are merely different dynamic modes of the same structural system. Gaits manifest themselves as different non-linear elastic oscillations that form distinct (yet connected) limit cycles that passively propel an animal or robot forward. It therefore implies that different phenomena observed in such systems should rely upon common models and principles. Below, we discuss a set of recent propositions on these concepts.

### 5.1. Metabolic Cost Model

Faraji et al. (2018) proposed a simple cost model to predict metabolic cost trends under general walking conditions. A 3D linear walking model (called 3LP) developed by Faraji and Ijspeert (2017) is used to predict swing and torso balance costs in the sagittal and frontal planes. The vertical collisional loss and recovery work missing in the 3LP model are included via a Center of Mass (CoM) velocity redirection cost. To account for walking with a non-zero amount of leg lift, a ground clearance cost is incorporated. A weight support cost is also added to account for the energy consumed by leg extensor muscles preventing the stance leg from collapsing under body weight. The resulting cost model is the sum of all four individual costs, scaled by a constant muscle efficiency to convert from positive mechanical work to metabolic input. To evaluate the model, a set of walking conditions from several studies are simulated, including variations in step frequency, step width, added mass, extra ground clearance, crouched walking and reduced gravity conditions. For example, while Donelan et al. (2001) found that the metabolic cost increases at a rate of 6.40 W/kg per meter squared of step width, a quadratic fit to the proposed model reveals a close rate of 5.21 W/kg per meter squared of step width. Overall, the proposed linear combination of four major costs can predict (within the data's 95% confidence interval) the metabolic cost of increasing step width and many other walking conditions. It also provides a detailed metabolic contribution of each component, which is valuable for improving or augmenting performance.

### 5.2. Bioinspired Insights

To realize principles of leg control for robust and economic locomotion over rough terrain, Daley (2017) focused on use of comparative biomechanics as a tool to derive insights into how mechanics and control are integrated to achieve agile, stable and economic locomotion. Birds serve as a useful bipedal animal model, because ground birds such as quail,fowl and ostriches use walking and running gaits that are similar in whole-body dynamics, limb trajectory and ground reaction forces to gaits of humans (Daley, 2018). To understand the principles of bipedal gait, it is essential to combine perspectives from biomechanics, sensorimotor control and engineering. Recent studies have focused on measuring movement biomechanics over simple terrain features, such as obstacle negotiation and single downward steps (Birn-Jeffery et al., 2014; Blum et al., 2014), gait transition dynamics of ostriches moving freely in an open field (Daley et al., 2016), and leg loading during kicking and locomotion in the snake-hunting secretary bird (Portugal et al., 2016). These studies provide insight into locomotor control strategies by comparing steady and transient movement tasks, and investigating potential trade-offs among factors such as speed, stability, robustness and economy.

Birn-Jeffery et al. (2014); Blum et al. (2014); Hubicki et al. (2015) compared bird running biomechanics to model predictions, using reduced-order models and trajectory optimization of bipedal locomotor dynamics, to directly test hypotheses about the priorities and mechanisms underlying bipedal locomotion control. Studies of obstacle and step negotiation revealed that running birds prioritize consistent leg loading (injury avoidance/safety), as the dominant control objective (Birn-Jeffery et al., 2014; Blum et al., 2014). These studies also revealed that the optimal leg trajectories to regulate leg loading (to maintain consistent forces) are similar to the optimal trajectories to minimize mechanical work. These findings highlight that control priorities for economy (minimal work) and safety (consistent forces) are closely aligned. Birds use a three-step recovery strategy over obstacles that reflects priority for economy and safety, but does not directly prioritize trajectory stabilization to maintain the nominal steady-state body center-of-mass dynamics. Running birds achieve stability through passive-dynamic mechanisms, so stability is not required as a direct target of actuation control, due to integration of passive-damping and (multi-articular) intrinsic compliance.These studies revealed that robustly stable and agile locomotion over uneven terrain can be achieved through a simple control strategy of prescribing a leg trajectory (and therefore foot landing conditions) to maintain desired leg loading as modeled by a simple point-mass spring-loaded inverted pendulum (SLIP) model. During stance, leg dynamics are asymmetric and consistent with work-minimizing control of an intrinsically damped leg model. These studies also found that the control strategy used by running birds was similar across many conditions, including unexpected (invisible) potholes, visible obstacles, single steps and multiple steps. Despite varying ability to anticipate the upcoming terrain, leg dynamics and control strategies remain consistent across contexts.

The principles from these studies have been implemented as control policies in the bipedal robot ATRIAS at Oregon State University (Hubicki et al., 2016b), resulting in robustly stable bipedal gaits that are dynamically similar to those of ostriches and humans. These studies have also provided insight into the similarities and differences between humans and birds as bipedal animals. Humans and birds share similar whole-body dynamics of walking and running gaits and similar ground reaction force patterns. However, humans and birds use different stride length and frequency characteristics that reflect their different leg morphology. Humans and birds also use different sensorimotor control strategies. Humans rely heavily on “cephalized” (brain-dominated) control, involving extensive learning and high reliance on predictive planning; however, these processes suffer from long control delays. In contrast, birds have specialized to more heavily rely on “spinalized” (spinal-cord dominated) control, primarily using spinal rhythm generation coupled to robustly stable intrinsic leg mechanics (Daley, 2018). Rehabilitation and control of prosthetic devices might benefit from bird-inspired control mechanisms to achieve robust stability using simple control algorithms and intrinsically stable leg mechanics.

### 5.3. Underlying Concept

Real-world applications require reasoning and decision-making higher-level control, for which complete perception data is necessary, in order to select suitable behavior and motion planning schemes. Patrick et al. (2018) discussed their research on planning for efficient reactive legged locomotion. Prior planning architectures for legged robots generally rely on either finding state trajectories with an on-line optimization process (see Feng et al., 2015; Kuindersma et al., 2016), or using a specific walking controller formula that allows for analytic approximations of the stance dynamics (see Arslan and Saranli, 2012; Englsberger et al., 2015). These reactive control methods are, however, poorly compatible with common robotic motion planning methods that rely on regulating the robot trajectory through its state space. To address this, Patrick et al. (2018) presented that an alternative way of planning legged locomotion is to plan through the *action space* of efficient *reactive legged behaviors* which is similar to the controllers shown by Hubicki et al. (2016a). The elements of this space consist of controllers for different periodic gaits and transient actions that can arbitrarily trade off efficiency and robustness. Motion planning using this action space makes the planner choose which behaviors the robot should execute at any given time. When disturbances occur, the behavior executing in the control layer takes immediate action to keep the robot from falling, and after some latency the planning layer can react by specifying a new plan that accounts for the new situation. As a result, the robot can be more robust to real-world disturbances, while also allowing the use of arbitrary energy-optimized gait controllers. However, to navigate through such a dynamic space, it is necessary to understand the underlying principles of locomotion.

Jonathan et al. (2017) adopted legged locomotion as a dynamical phenomenon, inspired by its analogy to a clock, to discuss the periodic attractor underlying most natural gaits.This expounds the dynamical phenomenon of legged locomotion including walking, running, skipping, hopping and jumping. However, it excludes decision-making and path-planning of the system, as well as balancing, gaits that maintain a center of pressure within a polygon of support, and slow one-foot-in-front-of-the-other gaits. This concept can be described as a cycle of energy between internal potential, gravitational, and kinetic energy, given compliant interaction^5^ renders bouncing due to discrete footsteps observed in bird experiments discussed above. The energy exchange cycle can therefore describe different locomotion modes, e.g., a specific shift in the cycle defines walking and running, and one can find damped oscillation a key to stabilization. It can then suffice for dynamics of legged locomotion and just relies on on-board proprioception similar to blind walking/running for humans, which requires only an inertial measurement unit (IMU).

Inspired by the evaluation of bird running over uneven terrain (Birn-Jeffery et al., 2014; Blum et al., 2014; Hubicki et al., 2015), the fundamental locomotor functionality of a legged system can be independent of environmental conditions such as light, and recovery from small terrain variation need not require exteroceptive sensory information and/or large processing. It is then important to integrate compliance carefully into the system. In addition to passive compliance that was discussed earlier in section 2, the employment of active compliance (impedance control) enables the replication of different (linear or non-linear) compliant behavior, in addition to the adjustment of damping for variable energy dissipation, inspired by damping/energy dissipation regulation of birds for robust stability in uneven terrain. Moreover, integration of compliance improves the Markov Decision Process (MDP) of expected response, especially when set-point trajectory is given through feed-forward approaches.

## 6. Summary

This paper reviewed recent progress in design and control of robotic systems from different perspectives, to indicate the current state of robotics in terms of energy efficiency, and to highlight solutions advancing this criterion. It includes a review of various robotic actuators exploiting compliance in series and in parallel for energy recycling, and discusses the importance of limb segmentation in design, dynamics analysis and control of dynamical systems. A set of energetically-established control approaches are explored, and compared with human behaviors/controls. In addition, a set of cutting-edge topics initiating new directions in locomotion are reported. Overall, we can briefly conclude that:

- It is essential to exploit compliance in actuation units, however, the choice of series or parallel implementation, and design of stiffness level, strictly depends on the application, which can be derived via a set of optimization approaches referred in this work. Nevertheless, in a generic conclusion, compliance in series can be more efficient when the stiffness is tuned properly.
- We need to pose the right optimal control problem first: *what is optimal?* careful steps are made from biological observations and inspirations, and then their verifications by developing robots exploiting the bio-inspired insights. The robot design is then optimized, and an optimal controller can be exploited as it renders the best feasible performance (optimality by principle).
- Limb segmentation plays a significant role in dynamic analysis of the robot, and the corresponding design and control. It is therefore necessary to avoid single pendulum simplification, and account for the correct number of limb linkages.
- Biological insights have shown that locomotion control need to mostly rely on proprioceptive data (IMU and force/torque sensing). In other words, robotic system should be able to blindly generate the basic pattern of robustly stable dynamic locomotion, while interoceptive data (vision sensing) used mainly for path planning and navigation. It therefore implies that a comprehensive understanding of locomotion principles is still incomplete.

## Author Contributions

All authors listed have made a substantial, direct and intellectual contribution to the work, and approved it for publication.

### Conflict of Interest Statement

The authors declare that the research was conducted in the absence of any commercial or financial relationships that could be construed as a potential conflict of interest.

## Footnotes

^1^Contents of this paper are mainly based on the presentations of IROS 2017 workshop titled “On the Energetic Economy of Robotics and Biological Systems: a challenging handicap to overcome”.

^2^Specific resistance is an index used to evaluate the energy efficiency of a mobile robot. It is defined as the ratio of the total energy consumption *E* for a travel of a distance *d* to the gravitational potential energy when the robot is lifted by the distance *d*, i.e., $\frac{E}{Mgd}$ with *M* and *g* representing the robot mass and gravitational acceleration; reported also by Kajita and Espiau (2008). This measure is equivalent to Cost of Transport (CoT).

^3^biological systems data is extracted from https://en.wikipedia.org/wiki/Muscle,Basalmetabolicrate,Humanbrain,Foodenergy

^4^We used the relation extracted in Robergs et al. (2007); however, we used twice the mass value, i.e., 55.14, 58.28, and 58.42 kg, as the formula was extracted from data corresponding to two legs (and significantly larger mass than the prototype), and then we divided the output power by two. Moreover, we subtracted a basic metabolic power of 80 W from the overall value as we compare with the prototype's electrical motor power.

^5^Due to the compliance of the locomotor and the environment/world, foot contact is a process, rather than an event, in which the tangential foot forces cannot be applied at the contact instance. The robot interactions with terrain should then be compliant since compliant oscillations are insensitive to external disturbances and can eliminate inelastic collisions. Interactions can accordingly be robust with respect to ground height and/or impedance changes resulting from the lack or imperfection of world models. Additionally, prevention from rigid collisions avoids large impact forces and unnecessary energy consumption.
